# Pupil contagion variation with gaze, arousal, and autistic traits

**DOI:** 10.1038/s41598-024-68670-7

**Published:** 2024-08-07

**Authors:** Martyna A. Galazka, Max Thorsson, Johan Lundin Kleberg, Nouchine Hadjikhani, Jakob Åsberg Johnels

**Affiliations:** 1 Gillberg Neuropsychiatry Centre, Institute of Neuroscience and Physiology, University of Gothenburg, Gothenburg, Sweden; 2 Division of Cognition and Communication, Department of Applied Information Technology, University of Gothenburg, Gothenburg, Sweden; 3 Section for Speech and Language Pathology, University of Gothenburg, Gothenburg, Sweden; 4 Athinoula A. Martinos Center for Biomedical Imaging, Massachusetts General Hospital, Harvard Medical School, Boston, MA USA; 5 Child Neuropsychiatric Clinic, Queen Silvia Children’s Hospital, Västra Götalandsregionen, Gothenburg, Sweden; 6 Department of Psychology, Stockholm University, Stockholm, Sweden; 7 Department of Clinical Neuroscience, Centre for Psychiatry Research, Karolinska Institute, Stockholm, Sweden

**Keywords:** Psychology, Human behaviour

## Abstract

Pupillary contagion occurs when one’s pupil size unconsciously adapts to the pupil size of an observed individual and is presumed to reflect the transfer of arousal. Importantly, when estimating pupil contagion, low level stimuli properties need to be controlled for, to ensure that observations of pupillary changes are due to internal change in arousal rather than the external differences between stimuli. Here, naturalistic images of children’s faces depicting either small or large pupils were presented to a group of children and adolescents with a wide range of autistic traits, a third of whom had been diagnosed with autism. We examined the extent to which pupillary contagion reflects autonomic nervous system reaction through pupil size change, heart rate and skin conductance response. Our second aim was to determine the association between arousal reaction to stimuli and degree of autistic traits. Results show that pupil contagion and concomitant heart rate change, but not skin conductance change, was evident when gaze was restricted to the eye region of face stimuli. A positive association was also observed between pupillary contagion and autistic traits when participants’ gaze was constrained to the eye region. Findings add to a broader understanding of the mechanisms underlying pupillary contagion and its association with autism.

## Introduction

Face-to-face social interactions consist of a complex interplay of verbal and nonverbal cues, with eye contact arguably constituting one of their fundamental aspects^1,2^. Starting early in development, eye contact affords critical interpersonal information about others’ interests and intentions^3^. As adults, we continue to rely on eye contact to initiate and structure interactions with others^4^. Interestingly, direct eye contact also facilitates a phenomenon less apparent to us in our daily exchanges, called pupillary contagion. Pupillary contagion refers to the process in which one’s pupil size adapts to the size of the pupils of another. This phenomenon has been demonstrated in different populations, across ages and even in chimpanzees^5–8^. Two primary aims motivated the current work. The first was to examine the extent to which pupillary contagion reflects physiological arousal in children. The second was to explore the extent to which pupillary contagion is associated with autistic traits. Across both these aims we examine if cueing gaze to the eyes would enhance the contagion effect and its association with autistic traits.

Regarding the first aim, empirical research has shown that increases in pupil size occur when we orient toward and attentionally engage with a person or an event^9,10^. These changes are modulated by increased cognitive effort, as well as by emotionally arousing stimuli^11,12^, presumably mirroring the activity of the locus coeruleus and the release of norepinephrine^13,14^. One interpretation is that pupil contagion is an indication that we are sensitive to the pupil size of others as indicators of their arousal and emotional states, and that changes in others’ pupil size evoke a corresponding response as well^6,15^. As members of a group-living species, it does make good evolutionary sense to be sensitive, responsive, and attuned to others’ states of arousal, and pupil contagion has been aptly described as a mechanism of “arousal transfer” between conspecifics^6,16^ that helps in predicting other’s behaviours^17^. However, this interpretation is not uncontested. The by far largest source of variation in pupil size is due to changes in luminosity, with pupil dilating in response to a decrease in light. And so, another potential—completely non-social—explanation for pupillary “contagion” has been proposed, which is more challenging to control in experimental setups. Specifically, because pupils are darker in color than the surrounding iris, the brightness of the eye region is reduced when images of dilated pupils are presented, potentially causing pupil dilation of the observing individual. However, luminosity-based artefacts are unlikely to account for pupillary contagion because the relationship between luminosity and pupil contagion appears inconsistent—some research studies report pupil contagion even when luminosity was equal between images^5^, whereas others do not^18, 19^, still others do not report pupil contagion even when luminosity is clearly different between stimuli^6^. Also, as demonstrated by Prochazkova and colleagues, pupil contagion does not occur in isolation, but seems to be coupled with increased activation in specific brain areas that are also active when engaging in the “theory of mind” reasoning^20,21^. Others have also shown increased activation of the amygdala, a region responsible for identifying biologically relevant events^22^ when observing dilated pupils^23,24^. Finally, pupillary contagion has been linked with ratings of trust^15^. Presumably, such specific brain activation and socio-emotional contextual factors would not occur if changes in the pupil were simply caused by luminance confounds.

In the current study, we probed the “arousal transfer” interpretation in a sample of children (oversampled for high autism traits) by examining whether contagion effects are evident in other indexes of arousal that are not affected by luminosity, such as changes in heart rate or skin conductance. To date, only a couple of studies linked pupil reaction with other indexes of sympathetic and parasympathetic activity in response to faces or isolated eye regions. One study with adult participants reported a positive correlation between pupil changes and heart rate, but not skin conductance response, following presentation of emotionally expressive faces^25^. In the context of contagion specifically, only one study to our knowledge has investigated the relationship between pupil response and other autonomic nervous system reactions. In this recent investigation, 5- and 6-month-old infants were presented either with cropped faces (i.e., oval shape without hair or ears) or isolated eye regions as the pupils on both types of stimuli were dilating and constricting. The study reports both pupil diameter response and skin conductance response to cropped faces, but only pupil response when presented with images of isolated eye regions^26^. Compared to these existing studies, here we made great efforts to develop stimuli set to be as natural as possible. Indeed, it has been objected^27^ that many stimuli used to probe the pupillary contagion effect in prior research have been overly non-natural in design, with e.g., grey pupils, geometrical shapes, cropped faces or only eye region. Here we examined pupillary contagion in response to emotionally expressive, color photographs of faces, in which the only adjusted parameter was the pupil size.

In regard to the second aim, it has been argued that exploring the bases of pupillary contagion is important from the perspective of atypical development. Autistic traits are behaviors and characteristics associated with autism spectrum disorder (ASD). While ASD is a formal neurodevelopmental diagnosis with a prevalence of about 1–2% of the population, research has shown that autistic traits are dimensionally represented in the general population as well, meaning that there is no clear demarcation between ASD cases and naturally occurring variation of these traits^28–30^. Behavioral-genetic data have confirmed that the dimensional nature of ASD and autistic traits are present also etiologically, with mainly heritable factors contributing to the variability^31^. Following a general trend in research^32,33^, here we follow this dimensional approach by exploring how the degree of autistic traits, rather than a binary diagnostic status, is associated with pupil contagion. Indeed, to ensure a large variation in autism traits in the current study, we oversampled our experimental cohort for higher autism traits, by including one third of participants/children with a formal ASD diagnosis. Importantly, individuals high on autistic traits exhibit challenges in communication impairments in recognizing and interpreting social cues, including those related to eye contact and facial expressions^34,35^ as well as repetitive and restricted behaviors and preoccupations. In terms of eye contact specifically, studies measuring gaze behavior have revealed that when looking at an image of another person’s face, individuals with autism, or those with high autistic traits, show significant reductions in gaze directed at the central part of the face, including the eyes^36–38^, although noted differences in experimental designs and technologies have revealed important variability in the findings^39^. However, despite some variability, restraining gaze to the eye region (using for example a fixation cross) may provide an important experimental control in research on social perception and processing^40^.

Only one previous study examined the pupil contagion effect in individuals with autism^41^. In that study, adolescents and adults with and without an autism diagnosis were presented with series of images of computer-generated faces whose pupils had been modified to reflect dilation and constriction. Pupil contagion to dilated pupils was evident in both groups, even though the autism group, at group level, gazed less towards the eye region. Interestingly, in the autism group, the amount of gaze to the eyes was negatively correlated with the magnitude of pupil contagion meaning that, on an individual level, autistic individuals who showed the largest pupil response to dilated pupils were those who looked particularly little to the eyes. The authors interpreted the results as lending support to a hyperarousal hypothesis of social processing alterations in autism; that is, low eye gaze might be a common way to handle a hyperarousal elicited by strong socio-affective stimuli, such as dilated pupils. This interpretation differs markedly from ideas of autism that conceives a lack of social motivation or detachment to the inner lives of others as the core deficit^42–44^. At the same time, lack of control over gaze patterns in^41^ meant that the correlation between amount of eye gaze and pupil response should be followed up, to directly explore the extent to which cueing gaze to the eye region affects the association between autistic traits and pupillary contagion. This is the approach we took in the current study.

In order to methodologically control gaze in experimental research, a frequently used approach is to cue gaze of the participants by directing it toward the eye region. In keeping with the hyperarousal account, several neuroimaging studies confirm that when presented with emotionally expressive faces, and importantly, when asked to focus on the fixation cross placed at the eye region, there is *increased* activation of several subcortical structures involved in negative emotional processing not only in individuals with autism^45–47^ but also in neurotypical individuals^48^. Experimental manipulation of gaze patterns toward the eyes has been found to enhance activation of pathways involved in stress and arousal during socioemotional processing; therefore, if pupil contagion is reflective of emotional arousal, we should see this effect across other indexes, especially when participants are asked to focus on the eye region.

We extended prior research by exploring the following hypotheses. In light of the interpretation that pupillary contagion reflects physiological arousal transfer between individuals, we examined evidence to support the existence of contagion across multiple indexes (*hypothesis 1a*) in response to naturalistic photographs. In relation to this, we also explored to what extent cueing gaze to the eye region enhances pupil contagion (*hypothesis 1b*). We then tested the hypothesis of a positive association between autistic traits and contagion as measured through pupil size, heart rate and skin conductance changes (*hypothesis 2a*)*.* We also explored if the effect might be especially clear when gaze was cued to the eye region (*hypothesis 2b*).

## Method

### Participants

A total of 67 children (30 girls) between the ages of 6 and 18 years (*M* = 129.9 months, *SD* = 38.5 months; or about 10 years) were recruited from the community via postings on social media platforms, including our clinic’s website, or postings sent directly to the parents living in close proximity to our lab by mail. One participant’s data was excluded from the data analysis due to lack of cooperation and interest, leaving 66 participants in total. Although the goal of the study was to explore autistic symptoms on a continuum, we aimed to effectively oversample for the presence of high autistic traits. Consequently, of the 66 children included in the study, 22 (9 girls) had a clinical diagnosis or were awaiting an evaluation (*n* = 1). Of the 22 participants, 19 had a primary diagnosis of autism, 2 general anxiety disorder and 1 ADHD.

#### Ethical approval and consent to participate

This research adhered to the principles of the Declaration of Helsinki and was approved by the Swedish Ethical Review Authority (dnr 2021-02996). Informed consent was taken from all the participants and their legal guardians for both study participation and publication.

### Stimuli

Color photographs of 16 children (8 girls, 8 boys) were selected from the Child Affective Facial Expression set (CAFE^49,50^) and the child subset of the Radboud Faces Database (RaFD^51^). Table 1 in the Supplementary materials provides more detail on the selected images. Of the 16 photographs, 8 depicted positive (happy) and 8 depicted negative (sad) emotional expression. Since prior research generally has not found any effect of specific emotionality on pupillary contagion^52^ we had no a priori hypothesis regarding emotional expressions but rather used these different valences for the sake of variability^41,53^. The 16 photographs were used to generate images depicting small and large pupils, presented in two conditions with or without a fixation cross.

**Table 1:** Spearman semi-partial correlation results of the differential response of pupil, heart rate and skin conductance to images with large and small pupils in two gaze conditions and AQ (z-scores), controlled for age and  amount of gaze.

Response	Gaze condition	*n*	*r*	CI 95%	*p* value
Pupil	NO-CROSS	65	− 0.11	[− 0.35, 0.14]	0.382
	CROSS	65	0.28	[0.03, 0.49]	0.028*
Heart rate	NO-CROSS	45	0.24	[− 0.06, 0.51]	0.116
	CROSS	45	− 0.08	[− 0.37, 0.23]	0.632
Skin conductance	NO-CROSS	63	− 0.15	[− 0.38, 0.11]	0.266
	CROSS	64	− 0.20	[− 0.43, 0.05]	0.114

*Statistical significance *p* < 0.05.

#### Pupil manipulation

**Figure 1:**
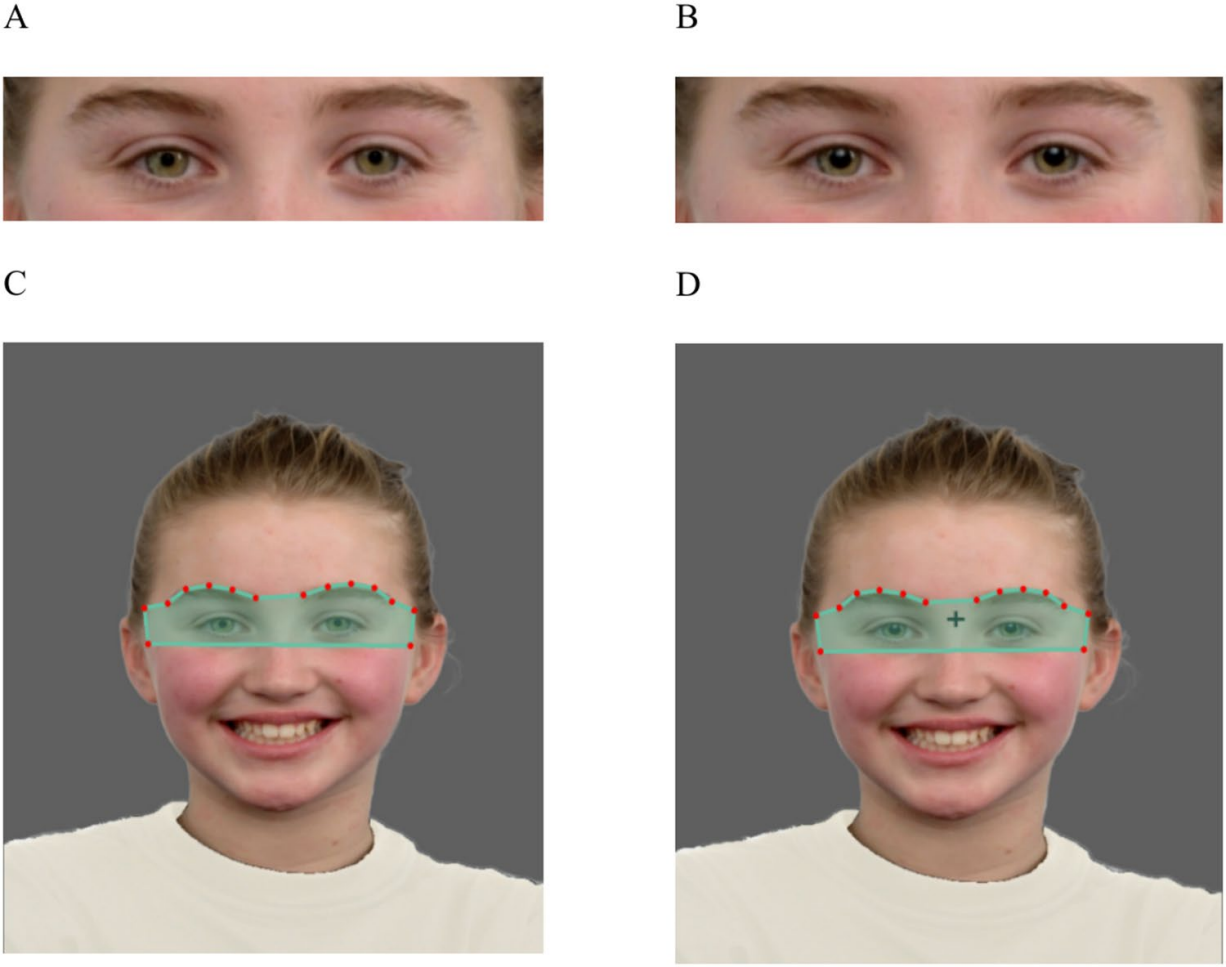
Top panel: A close-up of the stimuli with small (**A**) and large (**B**) pupils. Bottom panel: An example of the presented stimuli and of areas of interest for the eye region: NO-CROSS condition, small pupil (**C**), CROSS condition, large pupil (**D**). All stimuli were presented in the NO-CROSS and the CROSS condition for large and small pupils. Red dots display the identified facial landmarks, that formed the boundary of the area of interest.

To maintain experimental control and the most natural appearance of the photos, pupils in each photograph were manipulated to depict dilated pupils (large) and constricted pupils (small). Using Adobe Photoshop CS5.1, the pupils in each photograph were cropped out and resized. To create a small pupil condition, the pupils were resized to be approximately 30% (M = 29.8%, SD = 4.9%) of the width and approximately 40% (M = 38%, SD = 8.6%) of the height of the iris. To create the large pupil condition, the pupils were adjusted to approximately 50% (M = 54.4%, SD = 6.6%) of the width and approximately 70% (M = 71.3%, SD = 9.1%) of the height of the iris (see Table 2 in Supplementary materials). In addition to the pupil size adjustments, in some photographs, the color of the iris had to be made lighter in order to increase the visibility of the pupils (Fig. 1).

#### Luminance

When examining pupil contagion effect, luminance control is critical because changes in luminance may affect pupil reactions^18^. Luminance estimations, of the image as a whole as well as within eyes AOI, were generated in Python 3.86 (Table 2 Supplementary). To match luminance values between images with small and large pupils, we used Python script, following the example using Scikit-image for image equalization, available at their library documentation webpage (scikit-image, 2021). However, as noted previously by others^18^, it is important to recognize that it is not possible to fully match luminosity because a large pupil inevitably contains more darkness than a small one. Indeed, in our stimuli, although measures were taken to equalize the luminosity of each image in general, there were still slight (and arguably, unavoidable) differences in luminosity within the eye with slightly higher luminosity for the images depicting small pupils (*M*_*NO-CROSS*_ = 146.98 , *SD* = 13.62) than the large pupils (*M*_*NO-CROSS*_ = 146.6, *SD* = 13.56), but these were not statistically different (*W* = 42, *p* = 0.19, *n* = 16). Similar findings were in the CROSS condition with small pupils (*M*_*CROSS*_ = 145.78, *SD* = 13.33) and large pupils (*M*_*CROSS*_ = 145.54, SD = 12.74), *W* = 44, *p* = 0.23, *n* = 16 (see Table 2 in Supplementary materials for luminosity information).

### Apparatus

Experimental stimuli were presented on a 17-inch monitor with a screen resolution of 1280 × 1024 pixels (60 Hz refresh rate). For each photograph, the distance between the center of the eyes was calculated and at approximately 60 cm from the participant had an average visual degree of 5.3 degrees (ranging between 4.4 and 5.7 degrees for all images). Tobii T120 remote eye tracker was used to record eye movements and pupil size changes in a room where ambient light was controlled. Gaze was recorded at 120 Hz. A 9-point calibration procedure preceded the stimulus presentation.

Skin conductance response (SCR) was measured from the proximal phalanges of the index and the middle fingers of the non-dominant hand at a sampling rate of 128 Hz via the Shimmer3 GSR + Unit (Shimmer, MA, USA). Ag/AgCl electrodes with constant voltage (0.5 V) (Lafayette Instrument, IN, USA) were used.

Heart rate (HR) chest strap (Polar H10; Polar Electro Oy, Finland), with a detachable heart rate sensor which registered the heartbeat signal (R–R intervals) at 2 Hz. Performance of this device has been found to be consistent with the conventional 12-lead electrocardiographic (ECG) system^54^.

All indexes—gaze, pupil size, skin conductance measures and heart rate—were recorded during the experiment via the iMotions Biometric platform (iMotions A/S, Copenhagen, Denmark www.imotions.com) and data were synchronized using common system timestamps between these signals.

### Procedure

Upon arrival to the lab, participants and their guardians were informed about the procedure. Following consent, a trained researcher prepped the child’s fingers with alcohol wipe and child’s chest with warm water to increase registration of the sensors. The strap was then fastened around the participant’s chest at the level of the xiphoid (diaphragm). Skin conductance sensors were placed on the child’s fingers. Both registrations began and continued throughout the experiment. The study began with a baseline measure which was a 2 min resting period during which the participant was asked to sit comfortably in an armchair. This was done to reduce any arousing effects of study participation or being in a novel environment. Following the initial heart rate and skin conductance registration, the child was instructed to sit in front of the Tobii T120 eye tracker for the start of the stimulus presentation.

### Stimulus presentation

**Figure 2:**
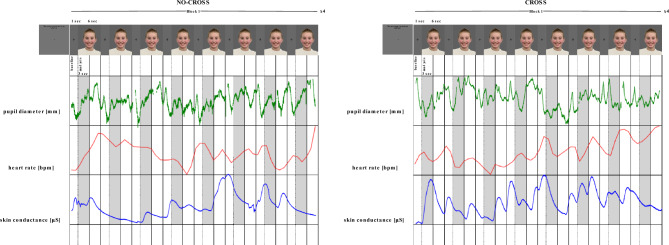
Schematic representation of the experimental procedure, illustrating the image presentation as well as time series used for analysis of pupil diameter, heart rate and skin conductance response. Images within the block differed but presentation of these is limited due to restriction on image rights.

For the illustration of the stimulus presentation please refer to Fig. 2. The experiment was a blocked design. All participants observed 8 blocks of trials (4 NO-CROSS and 4 CROSS) consisting of 8 different images presented for 6 s each and preceded by a 1 s inter-stimuli fixation cross. Stimulus presentation time was selected based on previous studies with similar populations^55–59^, as well as to account for potential variation in temporal structure of each response. A 10 s fixation cross preceded each block to allow return to baseline levels. Blocks differed by presented emotional expression (happy, sad) and pupil size of the image (large, small). The images within the block as well as the blocks themselves were presented in random order.

### Pre-processing of data and analysis

Data preprocessing and statistical analyses were performed using Python. The analysis period of 0–3 s post stimulus onset was chosen because it coincided with established timeframe of pupil contagion effect^6,26,41^. It is important to note that the aforementioned technologies used for assessing pupil size and galvanic skin response include signal noise, in the form of irregular fluctuations. These fluctuations may result from movement artefacts and variations in light, making it difficult to detect subtle response differences. Subtractive baseline correction is one approach that has been used to minimize the noise in the pupil size and skin conductance estimates^60^. For pupil and skin conductance estimates, 1 s fixation cross preceding stimulus presentation served as baseline measure. Subtractive baseline correction was not performed for the heart rate, which is designed to be comparatively stable for physical activity against this type of noise. In addition, block design allowed us to capture the cumulative effects resulting from repeated stimuli within each block for heart rate, while simultaneously correcting for the temporal fluctuations present in pupil size and skin conductance data.

#### Eye gaze

To account for a slight variation of presented images (different actors, slight head tilting, change within the facial features due to expressed emotions), an automatic generation of areas of interest (AOIs) for classification of gaze was used. Using a custom Python script, the area of interest covering both eyes (eyes AOI) was defined as a polygon boundary which was determined by the locations of automatically generated facial landmarks that included the eyebrow landmarks and the two upper cheek landmarks. To include the forehead, a half-ellipse was estimated (Fig. 1C, D). The choice of using relatively large AOI over the eye region is justified in view of well recognized challenge in assessing children, and especially those with a high degree of autistic traits with precision during eye tracking^61^.

In order to minimize data exclusion gaze coordinates, rather than fixations, were classified on whether they were within the eyes AOI, using a point-in-polygon problem solved by using the ray tracing method. Based on this classification, average of 3.2% (SD = 5.5%) of the trials was missing. For the final number of trials (*n* = 4106), missing data points were excluded from gaze position estimations. These accounted for 4.5% (SD = 12.9%) of total data points. The remaining *x* and *y* gaze coordinates from the left and right eyes were then interpolated linearly at a constant framerate of 120 Hz and averaged across eyes. The proportion of gaze within the eyes AOI was calculated by dividing the number of data points within the specified area by the total number of data points in the first 3 s of stimulus presentation for each participant. The code used in our analysis is available at the GitHub repository (https://github.com/thoraxmax/automaticAOIs.git), previously used in^62^.

#### Pupil response

Raw pupil data were exported from the iMotions software and pre-processed in Python. Pupil data with deviating dilation speed above 4 median absolute deviations, gaps larger than 10 consecutive samples, as well as 5 samples preceding and following the gap, were removed as described in^63^. Missing data, including eye blinks (*M*_CROSS_ = 20.1%; *SD*_CROSS_ = 10.8%; *M*_NO-CROSS_ = 22.4%, *SD*_NO-CROSS_ = 12.1%) were then linearly interpolated at the constant framerate of 120 Hz and filtered using a 4th-order Butterworth zero-lag low-pass filter at 4 Hz as a cut-off^64^. Filtered pupil data from the left and right eyes were averaged across both eyes. The average size of the pupils was estimated over a duration of 3 s. Finally, pupil values underwent baseline correction whereby the average pupil size estimated during 1 s of the preceding fixation cross was subtracted from the pupil size values during first 3 s of stimulus presentation. Finally, the baselined values were averaged across trials for each condition.

#### Skin conductance response

Following export from iMotions, calibrated conductance data were first linearly interpolated to a constant framerate of 120 Hz, and then high-pass filtered, 1st-order and with a cut-off of 0.05 Hz^65^ to isolate the phasic signal. Rapid deviations were then reduced by a 2nd-order zero-lag 5 Hz low-pass filter^66^. Similar to previous literature^25^ outliers above and below 2.5 SDs were removed. This exclusion criterion resulted in 38.1% (SD = 29.3%) missing trials in both conditions, with *n* = 2644 total trials that were included in the analysis.

For each trial, skin conductance response was estimated as the difference to average phasic signal during 1 s prior to stimulus onset and the largest SCR amplitude of the phasic signal in the first 3 s post stimulus onset. This difference was than averaged across trials for the resulting skin conductance response.

### Heart rate

Post-testing data inspection revealed that data from 18 participants (8 girls, 10 boys; 5 with diagnosis) were missing due to recording error. A major reason for this was that the belt did not fit snugly around the chest of some smaller-framed children, even though steps were taken to ensure it would remain in place. Processing of the remaining heart rate data began by removing outliers with criteria for RR-interval between 300 and 2000 ms^67^. The signal was then linearly interpolated to a constant framerate of 2 Hz. We then removed ectopic beats^68^ and estimated the heart rate (HR) as follows, *HR* = *6000/NN-intervals.* Furthermore, 4.9% (SD = 14.6%) of the trials had no registered data and could therefore not be included in the analysis, resulting in *n* = 2912 total trials. Finally, we estimated the average heart rate during the corresponding first 3 seconds from stimulus onset for each trial and calculated the average per condition per individual.

#### Autistic traits

Presence of autistic traits was assessed using the Autism Spectrum Quotient (AQ^69^) questionnaire. Originally designed for adults, this questionnaire has since been adapted to adolescents (Adolescent-AQ^70^) and children (AQ-Child^71^). The instrument consists of 50 statements related to functioning within different areas (social skills, attention switching, attention to detail, communication and imagination) corresponding to different subscales. The participants or their primary caregivers rate each statement on a 4-point scale ranging from ‘definitely agree’ to ‘definitely disagree.’ The resulting scores provide insight into the presence and severity of autistic traits, with higher scores indicating a greater degree of ASD-related characteristics. In the present study, the total AQ score, rather than specific subscales, was used to assess the global impairment because it has been found to have higher internal consistency (Cronbach’s alpha = 0.75 in typical sample and 0.84 in individuals with autism^72^. Three versions of the questionnaire were used in the present study. Parents of children between 6–11 years (*n* = 41) completed the AQ-Child questionnaire, while parents of children between 12–15 years (*n* = 20) completed the Adolescent-AQ questionnaire. Participants who were older than 16 years (*n* = 5), completed the Adult-AQ version themselves. Importantly, the AQ-Child and the Adolescent-AQ versions maintain the same underlying constructs as the self-reported Adult-AQ but use more age-appropriate language and examples. Scores above 76 on the AQ-Child, 24 on Adolescent-AQ and 29 on Adult-AQ have been found to be indicative of clinically significant autism traits. In order to equate the scores between the versions, each participant’s total AQ score was transformed into standard scores (z-score) based on means and standard deviations for age-appropriate standard reported in the following references: AQ-Child^71^; Adolescent-AQ^28,70^; Adult-AQ^69^. As expected, the present sample displayed a wide range of autistic traits, with z-scores ranging from -2.93 to 4.42 (*M* = 0.29, *SD* = 1.75), with higher values indicating more autism traits.

### Statistical analysis

In acknowledging the relatively small sample size, which was additionally affected by data loss on some biometric measures, potential noise, and because initial inspection indicated non-normal distribution of several included variables, we opted to conduct all analyses using more conservative non-parametric tests. Non-parametric tests are especially useful in our study because these tests do not make the same distributional assumptions as their parametric equivalent tests, do not rely on asymptomatic distributions for inferences that are not likely to hold for the small group size, and are more robust to outliers. To test the difference in arousal response within and across two conditions Wilcoxon signed-rank test were used. Spearman rank correlation coefficient (full and semi-partial) was used to examine the relationship between arousal responses and autistic traits.

### Permissions

The use of the materials in the present study has been approved by the authors of the materials. Images depicted in Figs. 1, 2 specifically were obtained from Radbound Faces Database, and we express our gratitude to Behavioural Science Institute of the Radbound University in Nijmegen, Netherlands, for granting us permission to use these images in this publication under terms and conditions provided. We have complied with all relevant copyright and ethical standards.

## Results

### Gaze to the eyes between constrained and unconstrained gaze conditions

The initial analysis examined the amount of gaze toward the eye area which included the fixation cross positioned between the eyes in the CROSS condition. This analysis revealed that, as expected, participants looked more to the eye region, when asked to focus their gaze there (*Mdn* = 74.7%, range: 6.6–97.1%) compared to when they were given no such instructions (*Mdn* = 51.2%, range: 12.0–90.7%), Wilcoxon signed-rank test, *W* = 91.0, *p* < 0.001, *n* = 66, *r* = − 0.80). The analysis of gaze also showed that older children looked more to the eyes in both conditions *r*_*NO-CROSS*_ (64) = 0.29, *p* = 0.017; *r*_*CROSS*_ (64) = 0.61, *p* < 0.001. There were no significant differences in the amount of gaze to images with large versus small pupils in either condition, *W*_*NO-CROSS*_ = 1020.0, *p* = 0.58, *r* = -0.067; *W*_*CROSS*_ = 1101.0, *p* = 0.98, *r* = − 0.003. Finally, the AQ score did not correlate with the amount of eye gaze in either condition (semi-partial Spearman), when accounting for age, *r*_NO-CROSS_ = -0.05. *p* = 0.87; *r*_CROSS_ = − 0.02, *p* = 0.19.

### Contagion in pupil, heart rate and skin conductance measure between large and small pupils

In order to address *hypotheses 1a* and *1b*, we examined whether there was evidence of changes in arousal when presented with the pictures of large versus small pupils, across pupil response, heart rate and skin conductance, in the two gaze conditions. To do so, we calculated a difference score between the three arousal measures when observing pictures depicting large and small pupils, resulting in three differential reactions such that,

$${\text{PR}}_{{{\text{diff}}}} = M\,{\text{pupil }}\,{\text{diameter}}_{{{\text{Large}}}} {-}M\,{\text{pupil}}\,{\text{ diameter}}_{{{\text{Small}}}}$$

$${\text{SCR}}_{{{\text{diff}} }} = M\,{\text{SCR}}_{{{\text{Large}}}} {-}M\,{\text{SCR}}_{{{\text{Small}}}}$$

$${\text{HR}}_{{{\text{diff}}}} \, = \, M \, \,{\text{HR}}_{{{\text{Large}}}} - M\,{\text{ HR}}_{{{\text{Small}}}}$$

Results indicate that in the NO-CROSS condition, none of the reactions were significantly above zero, meaning that there was no significant increase in response to images with large pupils. For descriptive statistics see Table 3 Supplementary.

**Figure 3:**
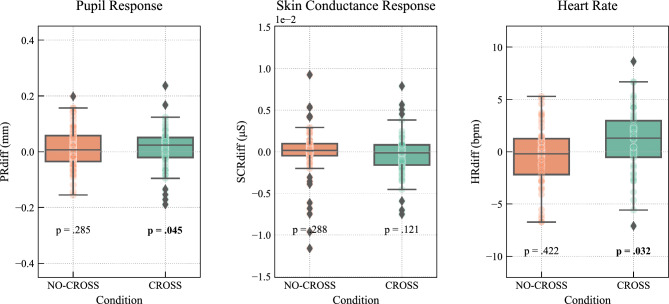
The difference in response to images of large and small pupils in pupil dilation, heart rate and galvanic skin response in the NO-CROSS and CROSS conditions. The box represents the quartiles and the median, while the whiskers show the data range. Flier points are values beyond the whiskers (diamonds) and all data points are shown as a colored scatter. Significances from paired Wilcoxon signed-rank tests are shown per conditions.

In the CROSS condition, there was a significant contagion effect in the pupil (*W* = 792.0, *p* = 0.045, *n* = 66, *r* = − 0.24) and heart rate (*W* = 345.0, *p* = 0.032, *n* = 46, *r* = − 0.31), but not skin response (Fig. 3) providing evidence for arousal transfer when examining pupil reaction (contagion) and heart rate changes, but not skin conductance (*hypothesis 1a*), and evidenced only when restricting participants’ gaze to the eye region (*hypothesis 1b*).

*Association between pupillary contagion and autistic traits.* Finally, we examined whether autistic traits correlate with the contagion response in the two gaze conditions. No significant correlation was evident between AQ scores and pupil contagion in the NO-CROSS condition, contradicting *hypothesis 2a*.

In the CROSS condition, however, pupil contagion response significantly correlated with autistic traits when age and amount of eye gaze were controlled for, *r*_*semi-partial*_(63) = 0.28, *p* = 0.028 (Table 1). In contrast, no associations between the AQ score responses in heart rate or skin response were found in either condition, providing partial support for hypothesis *2b*.

Finally, because of the potential effect luminosity differences have on pupil reactions, we examined the association between luminosity differences in the eyes AOI and pupil response (PR_diff_). We found that in the CROSS condition (Spearman’s *r* (14) = − 0.33, *p* = 0.21) and in the NO-CROSS condition (Spearman’s *r* (14) = − 0.28, *p* = 0.28), luminosity differences did not significantly correlate in differential pupil response, excluding this as a spurious cause for our observation.

## Discussion

The pupils of an observing individual dilate in response to images of dilated pupils in another individual—a reaction said to reflect transfer of arousal. The overarching aim of the present study was to understand the nature of this type of arousal. Specifically, we wished to better understand whether pupillary contagion reflects changes in physiological reactions along with pupil changes or whether this effect could be attributed to differences in luminosity. The present findings partially support the idea of pupillary contagion as a mechanism of arousal, as pupil size changes occurred not in isolation, but were coupled with comparable changes in heart rate. Importantly, these changes were evident only when children were instructed to focus on the eye region by looking on the fixation cross between the eyes of the presented faces, potentially suggesting that pupillary contagion may reflect reaction to pupils in the close visual periphery. Changes in the skin conductance were not observed.

It is important to reflect on these findings in terms of statistical strength and experimental design. First, the reported effect is relatively weak, and the small sample size means that the results should be interpreted with proper caution. Second, it is important to note that functionally, gaze restriction utilized in the present study is similar to paradigms used in studies that limit visual input to the eye region. The fact that we find an effect only in this condition, seems to suggest that pupil contagion may be evident only with limited visual input (such as presenting only the eye region), or to full but more controlled face stimuli (such as when presenting avatars and cropped faces that show only the central portions). This possibility may explain our failure to replicate our previous findings^41^ and more recent findings^26^ in the free-viewing condition. Realistic faces, with limited pupil manipulation such as those in the present study, may indeed effect magnitude of the pupillary contagion effect but other factors—including the age of the participants—may also play a role.

Why was the contagion effect not evident in the skin conductance response in our study? Of course, null findings are always difficult to interpret, especially in regard to measurement within a new paradigm. Of note is that we lost a significant amount of data (38.1%), mostly due to movement artefacts, or sensor contact loss with children’s much smaller fingers, raising the possibility of reduced statistical power. But the lack of changes might also be more substantive. One previous study examining pupil, heart rate and skin conductance^25^ to emotionally expressive human faces in adults (not in the context of contagion) was only able to find positive trial-by-trial correlations between pupil diameter and heart rate, but much like here, not with skin conductance. At the same time, a more recent finding in which electrodes were applied and secured to the infants’ foot sole did find comparable skin conductance changes to dilating pupils^26^. The existing variability in findings to which our study adds, indeed highlights the challenges of measuring and linking physiological reactions with vastly different temporal dynamics, stimuli and measurement methods. Finally, we consider the possibility that the lack of correlation between skin conductance response and pupil changes might have to do with low emotional reactivity in the observers. Indeed, when using images of high emotional valence such as those from the International Affective Picture System (IAPS), skin conductance changes are clearly more evident^73^. The naturalistic but static stimuli with pupil size manipulation used in the present study may have had an emotional valence that was not sufficient to evoke changes in skin conductance, a measure originally observed during startle or fear response^74^. And while skin conductance changes might be appropriate measures of physiological arousal, it may not be sufficiently sensitive in the context of contagion, although recent positive findings in infants cast shadow of a doubt on this interpretation in support of utilizing controlled and dynamic stimuli  using more age appropriate equipment. It would be worthwhile to more systematically address these concerns in the future.

The present study also explored the association between autistic traits, as measured by the AQ, and pupil contagion. We found that pupil contagion was positively correlated with autistic traits in the restricted gaze condition, but this was not the case for heart rate or for skin conductance. To the best of our knowledge, there is only one prior study^41^ exploring the link between autistic traits and pupillary contagion. The results from that study show that when presented with images of emotional faces, individuals with diagnosed autism exhibited a pupil response that was almost identical to those of non-autistic controls. Importantly, the magnitude of pupil contagion response was the same even though the ASD group looked significantly less at the eyes. In fact, within the autism group, there was a negative correlation between pupil response to images to large pupil versus small pupils and low fixations to the eye region. The remaining aspect of that study was whether gaze restriction would enhance emotional response. The current study contributes new insight in this regard, in confirming that gaze restriction positively effects pupil contagion in those with high autistic traits.

The present findings touch upon a larger theoretical discussion on the nature of the underlying differences in autism. Indeed, there is an ongoing discussion about whether low levels of social engagement and social perception should or should not be conceptualized as an insensitivity of autistic individuals to the inner lives of others^75^. Indeed, initial descriptions of autistic patients included statements about their ‘disregard for other’s opinion’^44^ later hypothesized to be attributed to differences in reward circuitry of the brain of autistic individuals^76^. An opposing hypothesis, developed in theoretical autism research^77^ and backed by empirical findings^45,78,79^ rather suggests that strong socio-affective stimuli, such as direct eye gaze, might instead be stressful and therefore are actively avoided among autistic individuals in order to down-regulate arousal. Empirically, recent neuroimaging studies confirm that when presented with emotionally expressive faces, and importantly, when asked to focus on the fixation cross placed at the eye region, there is increased activation of several subcortical structures involved in negative emotional processing in individuals with autism^45–47,73,76–79^. Arguably, such hyperarousal accounts seem also to better align with personal accounts of young adults with self-declared ASD as they describe their experiences with eye contact^80,81^. Experimental manipulation of gaze pattern toward the eyes has been found to differentially enhance activation of these subcortical pathways during socioemotional processing^45,46,79^. Here we find partial evidence in support of this hypothesis, in that we see a relationship between increase of autistic traits (in a population oversampled for autism diagnosis) related to pupil contagion, but  without a clear explanation for why a correlation between AQ and contagion effect was only evident in pupil response, but not in heart rate and skin conductance, and only when gaze was restricted to the eye region.

It should be noted there were missing and incomplete data resulting from technical issues during collection (heart rate) and presence of extreme values resulting from movement artefacts (skin conductance). Additionally, although statistically significant, the association between pupil reaction and autism is objectively weak (*r* = 0.28). In considering these aspects, the goal of future study efforts should therefore be to replicate and extend present findings in a larger group of individuals. Second, although the present study contributes to our understanding of the link between pupillary contagion and autistic traits, there remain significant avenues for future research in this regard, including the use of other measures to assess autistic traits beyond AQ. Finally, an unanswered question is whether the participants recognized the difference between the presented images. Although we did not consistently gather information on whether the participants were consciously aware of the differences in pupil size of the presented images, some older children expressed that they did, and one even noted it made her feel ‘uncomfortable.’ Future research could benefit from examining participants’ awareness of these differences in a more systematic way.

The present study explored pupil contagion and its relationship to emotional arousal. Results indicate that when participants viewed emotional faces with varying pupil sizes, changes in pupil size of the observer corresponded to changes in heart rate, but not skin conductance. This effect was more pronounced when participants focused on the eye region of the stimuli, highlighting the significance of gazing at the eye region. Increased pupil contagion was positively correlated with autistic traits in the restricted gaze condition, providing insights into processing differences in people with higher autism traits. Indeed, our study brings further evidence to the ongoing discussion within autism research about alterations in social engagement and perception, suggesting that socio-affective stimuli may be arousing for autistic (including high AQ) individuals, especially during direct eye contact.

### Supplementary Information

Supplementary Information.

Supplementary Tables.

## Data Availability

The raw data that support the findings of this study are available from the corresponding author upon reasonable request.
