# Supplementary material for: Pupil contagion variation with gaze, arousal, and autistic traits
Source: Sci Rep. 2024 Aug 7;14:18282. doi: 10.1038/s41598-024-68670-7 (PMC11306570; doi:10.1038/s41598-024-68670-7)
Supplement: Supplementary file 1 — Supplementary Information. [file 41598_2024_68670_MOESM1_ESM.docx]

**Supplementary analysis**

**Emotion**

Here we consider the magnitude of pupil response, heart rate and skin conductance response in each condition by emotional expression of the observed images (happy, sad). The analysis suggests no significant differences across indexes of arousal by presented emotion (all *p*s > 0.07).

**CROSS condition**

**Pupil response**

Wilcoxon signed-rank test, *W* = 1086.0, *p* = 0.90, *n* = 66

Mdn _Happy_ = 0.013, range = 0.614

Mdn _Sad_ = 0.029, range = 0.484

**Heart rate response**

Wilcoxon signed- rank test, *W* = 472.0, *p* = 0.461, *n* = 46

Mdn _Happy_ = 2.611, range = 22.75

Mdn _Sad_ = - 0.022, range = 28.85

**Skin response**

Wilcoxon signed-rank test, *W* = 759.0, *p* = 0.592, *n* = 60

Mdn _Happy_ = -6.577e-05, range = 0.018

Mdn _Sad_ = -5.805e-05, range = 0.022

**NO-CROSS condition**

**Pupil response**

Wilcoxon signed-rank test, *W* = 890.0, *p* = 0.169, *n* = 66

Mdn _Happy_ = -0.016, range = 0.561

Mdn _Sad_ = 0.032, range = 0.443

**Heart rate response**

Wilcoxon signed-rank test, *W* = 358.0, *p* = 0.072, *n* = 46

Mdn _Happy_ = -1.123, range = 13.93

Mdn _Sad_ = 0.722, range = 18.90

**Skin response**

Wilcoxon signed-rank test *W* = 769.0, *p* = 0.813, *n* = 59

Mdn _Happy_ = 6.392e-05, range = 0.0229

Mdn _Sad_ = 0.0002, range = 0.0218
