# Supplementary material for: Pupil contagion variation with gaze, arousal, and autistic traits
Source: Sci Rep. 2024 Aug 7;14:18282. doi: 10.1038/s41598-024-68670-7 (PMC11306570; doi:10.1038/s41598-024-68670-7)
Supplement: Supplementary file 2 — Supplementary Tables. [file 41598_2024_68670_MOESM2_ESM.docx]

**Supplementary Materials**

**Table 1 Supplementary.** Details regarding database origin, gender, age and ethnicity of the models used as a stimuli.

| **Model ID** | **Set** | **Gender** | **Age** | **Race/Ethnicity** |
| --- | --- | --- | --- | --- |
| AA-07 | CAFE | Male | 5 yrs | African American |
| AA-07 | CAFE | Female | 6.3 yrs | African American |
| AS-01 | CAFE | Male | 6.5 yrs | Asian |
| AS-01 | CAFE | Female | 3.9 yrs | Asian |
| EA-32 | CAFE | Male | 4.8 yrs | Caucasian/European American |
| EA-32 | CAFE | Female | 4.3 yrs | Caucasian/European American |
| EA-34 | CAFE | Male | 5.9 yrs | Caucasian/European American |
| EA-38 | CAFE | Male | 6.8 yrs | Caucasian/European American |
| EA-39 | CAFE | Female | 4.8 yrs | Caucasian/European American |
| EA-15 | CAFE | Female | 5.5 yrs | Caucasian/European American |
| Rafd090_39 | RaFD | Male |  | Caucasian |
| Rafd090_40 | RaFD | Male |  | Caucasian |
| Rafd090_44 | RaFD | Female |  | Caucasian |
| Rafd090-63 | RaFD | Male |  | Caucasian |
| Raf090-64 | RaFD | Female |  | Caucasian |
| Raf090-65 | RaFD | Female |  | Caucasian |

**Table 2 Supplementary:** Summary of experimental stimuli characteristics including testing condition, local luminosity levels and corresponding pupil size measurements.

| **IMAGE** | **Pupil Size** | **Condition** | **LUMINOSITY WHOLE IMAGE** | **Luminosity in eye region AOI** | **Pupil width (in px)** | **Pupil height (in px)** |
| --- | --- | --- | --- | --- | --- | --- |
| **H_F_Rafd090_64_LG.jpg** | large | NO-CROSS | 121.02 | 131.48 | 28 | 27 |
| **H_F_Rafd090_64_SG.jpg** | small | NO-CROSS | 120.36 | 131.08 | 17 | 15 |
| **H_F_Rafd090_65_LG.jpg** | large | NO-CROSS | 121.45 | 130.54 | 27 | 23 |
| **H_F_Rafd090_65_SG.jpg** | small | NO-CROSS | 121.88 | 130.44 | 21 | 19 |
| **H_F-AS-01_LG.jpg** | large | NO-CROSS | 122.70 | 172.13 | 67 | 65 |
| **H_F-AS-01_SG.jpg** | small | NO-CROSS | 123.21 | 173.03 | 34 | 32 |
| **H_F-EA-39_LG.jpg** | large | NO-CROSS | 121.70 | 157.65 | 33 | 33 |
| **H_F-EA-39_SG.jpg** | small | NO-CROSS | 120.93 | 159.69 | 28 | 28 |
| **H_M_Rafd090_39_LG.jpg** | large | NO-CROSS | 122.32 | 146.15 | 32 | 25 |
| **H_M_Rafd090_39_SG.jpg** | small | NO-CROSS | 122.32 | 146.86 | 16 | 15 |
| **H_M_Rafd090_40_LG.jpg** | large | NO-CROSS | 120.94 | 141.45 | 34 | 33 |
| **H_M_Rafd090_40_SG.jpg** | small | NO-CROSS | 119.99 | 143.31 | 20 | 16 |
| **H_M-AA-07_LG.jpg** | large | NO-CROSS | 121.89 | 147.36 | 56 | 54 |
| **H_M-AA-07_SG.jpg** | small | NO-CROSS | 122.83 | 148.80 | 35 | 45 |
| **H_M-EA-34_LG.jpg** | large | NO-CROSS | 122.56 | 151.40 | 30 | 30 |
| **H_M-EA-34_SG.jpg** | small | NO-CROSS | 121.94 | 150.51 | 26 | 24 |
| **S_F_Rafd090_44_LG.jpg** | large | NO-CROSS | 122.57 | 143.25 | 30 | 30 |
| **S_F_Rafd090_44_SG.jpg** | small | NO-CROSS | 122.51 | 144.32 | 14 | 14 |
| **S_F-AA-07_LG.jpg** | large | NO-CROSS | 123.88 | 128.81 | 70 | 68 |
| **S_F-AA-07_SG.jpg** | small | NO-CROSS | 123.33 | 129.96 | 31 | 28 |
| **S_F-EA-15_LG.jpg** | large | NO-CROSS | 122.35 | 138.89 | 64 | 61 |
| **S_F-EA-15_SG.jpg** | small | NO-CROSS | 122.19 | 137.36 | 26 | 27 |
| **S_F-EA-32_LG.jpg** | large | NO-CROSS | 119.80 | 131.47 | 62 | 54 |
| **S_F-EA-32_SG.jpg** | small | NO-CROSS | 119.70 | 131.79 | 32 | 28 |
| **S_M_Rafd090_63_LG.jpg** | large | NO-CROSS | 122.61 | 140.70 | 27 | 23 |
| **S_M_Rafd090_63_SG.jpg** | small | NO-CROSS | 122.65 | 141.76 | 12 | 12 |
| **IMAGE (cont’d)** | **Pupil Size** | **Condition** | **Luminosity**  **whole image** | **Luminosity**  **eye region AOI** | **Pupil width**  **(in px)** | **Pupil height**  **(in p**x**)** |
| **S_M-AS-01_LG.jpg** | large | NO-CROSS | 123.45 | 170.38 | 25 | 26 |
| **S_M-AS-01_SG.jpg** | small | NO-CROSS | 123.60 | 170.40 | 46 | 43 |
| **S_M-EA-32_LG.jpg** | large | NO-CROSS | 121.15 | 154.81 | 45 | 44 |
| **S_M-EA-32_SG.jpg** | small | NO-CROSS | 121.41 | 153.11 | 32 | 31 |
| **S_M-EA-38_LG.jpg** | large | NO-CROSS | 121.11 | 166.85 | 46 | 43 |
| **S_M-EA-38_SG.jpg** | small | NO-CROSS | 121.20 | 166.90 | 20 | 18 |
| **H_F_Rafd090_64_LGC.jpg** | large | CROSS | 120.98 | 131.99 | 28 | 27 |
| **H_F_Rafd090_64_SGC.jpg** | small | CROSS | 120.32 | 131.99 | 17 | 15 |
| **H_F_Rafd090_65_LGC.jpg** | large | CROSS | 121.39 | 129.13 | 27 | 23 |
| **H_F_Rafd090_65_SGC.jpg** | small | CROSS | 121.37 | 129.50 | 21 | 19 |
| **H_F-AS-01_LGC.jpg** | large | CROSS | 122.67 | 169.93 | 67 | 65 |
| **H_F-AS-01_SGC.jpg** | small | CROSS | 123.18 | 172.57 | 34 | 32 |
| **H_F-EA-39_LGC.jpg** | large | CROSS | 121.65 | 156.38 | 33 | 33 |
| **H_F-EA-39_SGC.jpg** | small | CROSS | 120.89 | 158.52 | 28 | 28 |
| **H_M_Rafd090_39_LGC.jpg** | large | CROSS | 122.27 | 145.40 | 32 | 25 |
| **H_M_Rafd090_39_SGC.jpg** | small | CROSS | 122.27 | 145.64 | 16 | 15 |
| **H_M_Rafd090_40_LGC.jpg** | large | CROSS | 120.90 | 140.48 | 34 | 33 |
| **H_M_Rafd090_40_SGC.jpg** | small | CROSS | 119.95 | 142.12 | 20 | 16 |
| **H_M-AA-07_LGC.jpg** | large | CROSS | 121.86 | 147.39 | 56 | 54 |
| **H_M-AA-07_SGC.jpg** | small | CROSS | 123.38 | 145.46 | 35 | 45 |
| **H_M-EA-34_LGC.jpg** | large | CROSS | 122.51 | 149.33 | 30 | 30 |
| **H_M-EA-34_SGC.jpg** | small | CROSS | 121.90 | 149.62 | 26 | 24 |
| **S_F_Rafd090_44_LGC.jpg** | large | CROSS | 122.53 | 143.34 | 30 | 30 |
| **S_F_Rafd090_44_SGC.jpg** | small | CROSS | 122.47 | 143.58 | 14 | 14 |
| **S_F-AA-07_LGC.jpg** | large | CROSS | 123.86 | 129.55 | 70 | 68 |
| **S_F-AA-07_SGC.jpg** | small | CROSS | 123.31 | 127.35 | 31 | 28 |
| **S_F-EA-15_LGC.jpg** | large | CROSS | 122.33 | 136.67 | 64 | 61 |
| **S_F-EA-15_SGC.jpg** | small | CROSS | 122.17 | 138.07 | 26 | 27 |
| **IMAGE (cont’d)** | **Pupil Size** | **Condition** | **Luminosity whole image** | **Luminosity in eye region AOI** | **Pupil width**  **(in px)** | **Pupil height**  **(in p**x**)** |
| **S_F-EA-32_LGC.jpg** | large | CROSS | 119.77 | 131.65 | 62 | 54 |
| **S_F-EA-32_SGC.jpg** | small | CROSS | 119.67 | 130.40 | 32 | 28 |
| **S_M_Rafd090_63_LGC.jpg** | large | CROSS | 122.57 | 140.58 | 27 | 23 |
| **S_M_Rafd090_63_SGC.jpg** | small | CROSS | 122.61 | 140.34 | 12 | 12 |
| **S_M-AS-01_LGC.jpg** | large | CROSS | 123.41 | 168.25 | 25 | 26 |
| **S_M-AS-01_SGC.jpg** | small | CROSS | 123.56 | 168.15 | 46 | 43 |
| **S_M-EA-32_LGC.jpg** | large | CROSS | 121.11 | 153.07 | 45 | 44 |
| **S_M-EA-32_SGC.jpg** | small | CROSS | 121.37 | 153.48 | 32 | 31 |
| **S_M-EA-38_LGC.jpg** | large | CROSS | 121.05 | 163.12 | 46 | 43 |
| **S_M-EA-38_SGC.jpg** | small | CROSS | 121.14 | 163.32 | 20 | 18 |

**Table 3 Supplementary.** Median (Mdn) and range values for Skin Conductance, Heart Rate, Pupil Diameter and Eye Gaze across CROSS and NO-CROSS conditions to images with Large, Small pupils and the difference (Diff) value between the two.

|  |  | ***Skin Conductance*** | | ***Heart Rate*** | | ***Pupil Diameter*** | | ***Eye Gaze*** | |
| --- | --- | --- | --- | --- | --- | --- | --- | --- | --- |
| **Condition** | **Stimuli** | **Mdn** | **range** | **Mdn** | **range** | **Mdn** | **range** | **Mdn** | **range** |
| **CROSS** | **Diff** | -0.000124 | 0.015398 | 1.318852 | 15.721166 | 0.022402 | 0.425984 | -0.597222 | 91.836806 |
|  | **Large** | 0.000213 | 0.014015 | 85.639438 | 42.786223 | -0.093107 | 0.423462 | 74.496528 | 88.003472 |
|  | **Small** | 0.000210 | 0.016360 | 83.987339 | 44.343940 | -0.094772 | 0.472768 | 73.663194 | 96.420139 |
| **NO-CROSS** | **Diff** | 0.000178 | 0.020845 | -0.182673 | 12.006549 | 0.007290 | 0.354161 | 1.284722 | 58.993056 |
|  | **Large** | 0.000589 | 0.009847 | 85.221581 | 40.317557 | -0.087865 | 0.456271 | 49.652778 | 79.618056 |
|  | **Small** | 0.000477 | 0.017242 | 85.710408 | 38.344275 | -0.112221 | 0.457427 | 51.310764 | 77.725694 |
